# Crimean-Congo Hemorrhagic Fever Disease Awareness Scale: Scale Development, Validity, and Reliability Study

**DOI:** 10.2196/93724

**Published:** 2026-09-14

**Authors:** Gülay Yilmazel, Ayşe Çalmaz, Emre Keleş

**Affiliations:** 1 Application and Research Center for Occupational Diseases & Faculty of Health Sciences, Hitit Üniversitesi, Gazi St, Çorum, 19000, Türkiye, 90 3642230730, 90 3642230733; 2 Hitit University Iskilip MYO, Çorum; 3 Department of Public Health Nursing, Faculty of Health Sciences, Hitit University, Çorum

**Keywords:** awareness, scale development, psychometric validation, validity, reliability, public health, Crimean-Congo hemorrhagic fever

## Abstract

**Background:**

Crimean-Congo Hemorrhagic Fever (CCHF) is a life-threatening, tick-borne, viral disease with substantial public health importance, particularly in endemic regions. Because no licensed vaccine or specific antiviral treatment is currently available, improving public awareness remains a key strategy for preventing transmission and promoting early diagnosis. However, no standardized instrument has been available to comprehensively assess CCHF awareness among adults.

**Objective:**

This study aimed to develop and psychometrically evaluate the Crimean-Congo Hemorrhagic Fever Disease Awareness Scale (CCHF-DAS) for use among adults.

**Methods:**

This cross-sectional methodological study was conducted between March and June 2025 among 293 adults recruited from 2 family health centers in Türkiye. An initial 29-item scale was developed following a comprehensive literature review and expert evaluation using the Davis technique. A pilot study involving 45 individuals assessed item clarity and comprehensibility. Construct validity was examined using exploratory factor analysis (EFA) and confirmatory factor analysis (CFA). EFA was performed to determine the underlying factor structure of the scale, and CFA was subsequently conducted to evaluate the fit of the proposed factor structure. Reliability was evaluated using Cronbach α, split-half reliability, composite reliability, average variance extracted (AVE), McDonald ω, and item-total correlations. The Harman single-factor test was performed to assess common method variance.

**Results:**

Content validity indices indicated excellent expert agreement for all items (Item Content Validity Index=1.00). EFA resulted in a unidimensional 25-item structure explaining 35.66% of the total variance. The Kaiser-Meyer-Olkin value was 0.912, and the Bartlett test was significant (*χ*²_4_=3385.4; *P*<.001), supporting sample adequacy. CFA demonstrated acceptable model fit (*χ*²_4.15_/df=4.15, root mean square error of approximation=0.075; standardized root mean square residual=0.072; goodness-of-fit index=0.95; adjusted goodness-of-fit index=0.94; comparative fit index=0.97). The scale demonstrated excellent internal consistency (Cronbach *α*=0.916; McDonald ω=0.931; composite reliability=0.931), although the AVE (0.356) was below the recommended threshold. Split-half reliability coefficients (Spearman-Brown=0.899; Guttman=0.898) further supported reliability. The Harman single-factor test indicated that common method bias was unlikely to be substantial.

**Conclusions:**

The CCHF-DAS is the first validated instrument specifically designed to assess adult awareness of CCHF. The scale demonstrated satisfactory construct validity and excellent reliability, supporting its use in epidemiological research, public health surveillance, and evaluation of educational interventions. Further validation in larger, culturally diverse populations and independent samples is recommended.

## Introduction

Crimean-Congo Hemorrhagic Fever (CCHF) is a tick-borne, viral, zoonotic disease caused by an enveloped, single-stranded RNA virus belonging to the *Nairovirus* genus of the *Bunyaviridae* family. The virus is primarily transmitted through ticks of the *Hyalomma* genus and is responsible for severe viral hemorrhagic fever outbreaks, with reported case-fatality rates ranging from 10% to 40% worldwide [1]. Owing to its high mortality rate, broad geographic distribution, and potential for epidemic spread, CCHF represents a major and persistent public health threat in endemic regions.

In Türkiye, CCHF first emerged as a public health concern in 2002, with confirmed diagnoses reported in 2003 following case clusters in Tokat Province. Since then, the disease has shown sustained endemicity, particularly in Central and Eastern Anatolia and the western and southern parts of the Black Sea Region. According to official data from the Ministry of Health of the Republic of Türkiye, 11,041 CCHF cases and 528 related deaths were reported between 2002 and 2018, corresponding to a case-fatality rate of 4.78% [2-5]. The disease is associated with a reported global case-fatality rate ranging from 10% to 40%, although mortality varies considerably across countries depending on factors such as health care infrastructure, surveillance systems, early diagnosis, and access to supportive treatment. This lower national fatality rate is thought to reflect improvements in disease surveillance, prompt diagnosis, experienced clinical management, and supportive care. Nevertheless, CCHF continues to represent a significant public health challenge because of its epidemic potential, wide geographic distribution, and lack of a licensed vaccine or specific antiviral therapy. Human infection occurs primarily through the bite or crushing of infected *Hyalomma* ticks, as well as through direct contact with the blood or bodily fluids of infected humans or animals [1,6,7]. A wide range of domestic and wild animals—including cattle, sheep, and goats—serve as amplifying hosts for the virus. Although these animals are typically asymptomatic, they can sustain viral replication, thereby facilitating virus circulation among ticks and increasing the risk of zoonotic transmission to humans through occupational or environmental exposure [2,8].

The epidemiology of CCHF is strongly influenced by ecological and environmental factors. Climate change, migratory bird patterns, and uncontrolled animal movement have been shown to contribute significantly to the expansion of tick habitats and the geographic spread of the virus [8,9]. These dynamics increase the likelihood of human–vector contact and complicate disease control efforts, particularly in rural and agricultural settings.

Following an incubation period of approximately 3 to 7 days, infected individuals typically present with nonspecific symptoms such as sudden-onset fever, headache, myalgia, arthralgia, nausea, vomiting, fatigue, and anorexia. In severe cases, the disease may progress to hemorrhagic manifestations involving the skin, mucous membranes, and internal organs, substantially increasing the risk of fatal outcomes [2,3].

Despite its high fatality rate and expanding geographic range, there is currently no specific antiviral treatment or licensed vaccine available for CCHF. For this reason, the World Health Organization has classified CCHF as a high-priority pathogen requiring urgent research and strengthened public health interventions [1,10]. In the absence of effective medical countermeasures, prevention strategies rely heavily on minimizing exposure risk through personal protective behaviors and public awareness initiatives [5].

Preventive measures recommended for individuals living in or traveling to endemic areas include wearing light-colored protective clothing, tucking trousers into socks, performing thorough body checks after potential tick exposure, and ensuring prompt and safe tick removal without direct hand contact [1,5]. Individuals are also advised to monitor for symptoms such as fever, headache, and malaise for up to 10 days following tick removal to facilitate early diagnosis and treatment [10].

Certain population groups are at disproportionately higher risk for CCHF, including individuals residing in endemic regions, agricultural and livestock workers, veterinarians, hunters, soldiers, campers, and health care workers exposed to infected patients or biological materials [1,4]. Despite these well-established risk factors and the disease’s significant epidemic potential, CCHF remains relatively neglected in terms of systematic public awareness assessment and preventive education.

In the absence of an effective vaccine, raising awareness of CCHF risk factors and preventive behaviors constitutes the most critical strategy for reducing infection rates and disease-related mortality. Public awareness not only supports primary prevention but also facilitates early recognition and timely medical intervention, particularly during seasonal peaks of disease transmission. However, a review of national and international literature indicates that no validated measurement tool currently exists to assess adults’ awareness of CCHF and its prevention.

Within this context, the present study aims to address this critical gap by developing a valid and reliable awareness scale to assess adults’ knowledge, perceptions, and preventive behaviors related to CCHF at both national and international levels.

## Methods

### Study Design and Participants

This study was conducted using a cross-sectional methodological design aimed at developing and validating a disease-specific awareness scale. The study was reported in accordance with the STROBE (Strengthening the Reporting of Observational Studies in Epidemiology) guidelines to ensure transparency and methodological rigor.

### Study Setting and Participant Recruitment

The study was conducted in 2 government-affiliated family health centers located in the city center of Çorum, Türkiye. These centers provide comprehensive primary health care services to approximately 13,000 registered individuals from urban and peri-urban communities, including preventive, curative, maternal and child health, vaccination, and chronic disease follow-up services. Participant recruitment was carried out between March and June 2025 using a consecutive sampling approach. During the data collection period, all adults attending either family health center for any health care–related reason (eg, routine examinations, prescription renewal, vaccination, preventive health services, or follow-up appointments) were systematically approached by a trained researcher while waiting for their consultation. Individuals were first screened for eligibility according to the predefined inclusion and exclusion criteria. Eligible individuals received a standardized explanation of the study objectives and procedures, and those who agreed to participate provided written informed consent before enrollment.

Recruitment was conducted on all working days throughout the study period to minimize selection bias. Each participant completed the questionnaire only once through a face-to-face interview conducted in a private area of the health center to ensure confidentiality and reduce external influence on responses. Recruitment continued consecutively until the predetermined target sample size was reached. Individuals who declined participation or did not meet the eligibility criteria were not replaced using selective sampling.

### Ethical Considerations

Ethical approval for the study was obtained from the Hitit University Non-Interventional Research Ethics Committee (Approval 2024‐22).

#### Informed Consent

All procedures were carried out in accordance with the principles of the Declaration of Helsinki. Written informed consent was obtained from all participants before enrollment. Before participation, eligible individuals received standardized verbal and written information regarding the study objectives, procedures, voluntary nature of participation, confidentiality of the collected data, and their right to withdraw from the study at any time without any consequences. Only participants who provided written informed consent were included in the study.

#### Privacy and Confidentiality

To protect participants’ privacy, no personally identifiable information (eg, names, national identification numbers, telephone numbers, or addresses) was collected during the study. Each questionnaire was assigned a unique study identification code, and all data were deidentified prior to analysis. The dataset was accessible only to the research team and was stored on password-protected computers in accordance with institutional data security procedures. Study findings are reported only in aggregate form, ensuring that individual participants cannot be identified directly or indirectly.

#### Participant Compensation

Participants did not receive any financial compensation, incentives, gifts, or reimbursement for their participation in the study. Participation was entirely voluntary.

### Data Collection Instruments

Data were collected using 2 instruments: a personal information form and the Crimean-Congo Hemorrhagic Fever Disease Awareness Scale (CCHF-DAS), which was developed by the researchers based on a comprehensive review of the relevant literature.

The personal information form consisted of 15 items designed to collect sociodemographic and health-related characteristics of the participants. The form included questions on age, gender, educational level, marital status, long-term place of residence, presence of chronic diseases, personal history of tick bites, tick exposure among relatives or close contacts, perceived occupational risk, and prior training or education related to CCHF. Participants were asked whether they had ever (during their lifetime) been bitten by a tick. Among those reporting a lifetime history of tick bites, information on the method of tick removal and hospital attendance and treatment was collected as lifetime history and was not linked to a specific tick bite episode. Participants were asked whether they had ever received any education or information regarding CCHF. This variable included both formal and informal educational activities, regardless of the setting. Participants who responded positively were additionally asked to identify the primary source of this information (health care professionals, media, or other sources).

### Development Process of the CCHF-DAS

#### Overview

The CCHF-DAS was developed specifically for this study to assess adults’ awareness of CCHF and preventive measures. The scale was originally developed in Turkish, and its English version is provided in Multimedia Appendix 1. The scale development process included item generation, content validity assessment, pilot testing, and psychometric evaluation.

#### Item Generation

Following the identification of the research objective, a comprehensive literature review was conducted to identify existing studies on CCHF-related knowledge, awareness, attitudes, and preventive practices. Searches were performed in the Web of Science, Scopus, PubMed, and ScienceDirect databases using the keywords “Crimean-Congo Hemorrhagic Fever,” “vector-borne disease,” “knowledge,” “attitude,” and “practice.” As no disease-specific psychometric tool addressing CCHF awareness was identified, the development of a new measurement instrument was deemed necessary.

An initial pool of 29 items was generated based on the literature review. Items were structured using a 5-point Likert-type scale ranging from “Strongly agree” to “Strongly disagree.” The initial item pool was developed to comprehensively represent 4 conceptual content domains of CCHF awareness—disease transmission, symptoms, risk factors, and preventive behaviors. However, these domains were conceptualized as complementary components of a single overarching construct, namely overall CCHF disease awareness, rather than as independent latent dimensions. Therefore, exploratory factor analysis (EFA) was conducted to determine whether the items reflected a unidimensional measurement structure.

#### Content Validity

Content validity of the draft CCHF-DAS was evaluated using expert review and the Davis technique. A total of 5 academic experts—2 from infectious diseases and 3 from public health—were consulted to assess the relevance, clarity, and cultural appropriateness of each item. Experts rated each item on a 4-point scale (1=not appropriate, 2=partially appropriate, 3=fairly appropriate, and 4=completely appropriate).

The Item Content Validity Index (I-CVI) was calculated for each item, with a threshold of ≥0.80 considered acceptable. All items achieved an I-CVI value of 1.00; therefore, no items were removed at this stage. Minor revisions were made based on expert feedback to improve clarity and wording.

#### Pilot Testing

The 29-item draft version of the CCHF-DAS was pilot tested with 45 individuals (25 women and 20 men) who shared similar characteristics with the target study population. Face-to-face interviews were conducted to assess item clarity, comprehensibility, and completion time. Participants were asked to review each item carefully and provide feedback regarding its meaning and ease of understanding. The average time required to complete the scale was recorded. Based on positive feedback and the absence of major comprehension issues, the scale development process proceeded to the main study. Participants involved in the pilot study were excluded from the final sample.

### Sample Size

In scale development studies, it is recommended that the sample size be at least 10 times the number of items included in the scale. Accordingly, given the 29-item draft version of the CCHF-DAS, a minimum sample size of 290 participants was required [11]. Additionally, methodological guidelines suggest that a sample size of at least 200 participants is sufficient for conducting EFA and confirmatory factor analysis (CFA). Taking potential data loss into account, the target sample size was set at 300 participants, and data were ultimately collected from 293 individuals. Inclusion criteria were being aged 18 years or older; being registered at the participating family health centers; having no visual, hearing, speech, or diagnosed psychiatric disorders; being able to communicate in Turkish; and providing informed consent. Exclusion criteria were age less than 18 years; inability to communicate in Turkish; cognitive impairment; visual, hearing, or speech disorders that could interfere with questionnaire completion; and refusal to participate. Medical education and previous CCHF infection were not assessed and therefore were not used as exclusion criteria.

### Data Analysis

Data were analyzed using SPSS (IBM Corp) for Windows version 22.0 and LISREL version 8.80. Descriptive statistics, including frequencies, percentages, minimum and maximum values, means, and SDs, were calculated to summarize the characteristics of the study sample. Content validity was assessed using the Davis technique. The suitability of the data for EFA was evaluated using the Kaiser-Meyer-Olkin (KMO) measure of sampling adequacy and Bartlett test of sphericity. Factor retention was initially evaluated using the Kaiser criterion (eigenvalues >1.0), examination of the scree plot, factor loading patterns, and conceptual interpretability of the extracted factors. Although the Kaiser criterion identified multiple factors with eigenvalues greater than 1.0, the final number of retained factors was determined by considering statistical evidence together with the theoretical framework of the scale. Preference was given to the most parsimonious and conceptually interpretable solution.

EFA was first performed to identify the underlying factor structure of the scale. Subsequently, CFA was conducted to evaluate the goodness-of-fit of the resulting measurement model. Although the use of an independent validation sample is recommended, EFA and CFA have also been applied to the same dataset in scale development studies when obtaining an additional sample is not feasible [12,13]. Factor retention was initially evaluated using the Kaiser criterion (eigenvalues >1.0), examination of the scree plot, factor loading patterns, and conceptual interpretability of the extracted factors. Although factors with eigenvalues greater than 1.0 were identified, the final number of retained factors was determined by considering both statistical evidence and theoretical coherence. Preference was given to the most parsimonious and conceptually interpretable solution. Model fit was evaluated using multiple goodness-of-fit indices, including the chi-square to *df* ratio (*χ*²/df), goodness-of-fit index (GFI), adjusted goodness-of-fit index (AGFI), comparative fit index (CFI), root mean square error of approximation (RMSEA), and standardized root mean square residual (SRMR). The structural relationships between observed variables and latent constructs were illustrated using a path diagram. Internal consistency and reliability of the scale were assessed using Cronbach α coefficient, item-total correlation coefficients, split-half reliability, composite reliability (CR), average variance extracted (AVE), and McDonald ω coefficient. Because the final scale demonstrated a unidimensional structure, discriminant validity using the Fornell-Larcker criterion was not applicable, as this method requires at least 2 distinct latent constructs for comparison [14].

In this study, variance inflation factor was not reported because the present study was not based on a regression or PLS-SEM model, where variance inflation factor is routinely used to assess multicollinearity. To evaluate the potential influence of common method variance (CMV), the Harman single-factor test was performed [15-17].

## Results

### Demographic Characteristics of the Participants

The demographic characteristics of the participants are summarized in Table 1. The mean age of the participants was 38.76 (SD 12.92) years. More than half of the participants were female (173/293, 59.0%), and approximately one-third had completed high school education (99/293, 33.8%). The majority of participants were married (196/293, 66.8%) and resided in the city center (250/293, 85.3%). Regarding employment status, 41.3% (121/293) of the participants were employed by an employer. Most participants reported having no chronic disease (231/293, 78.8%). Tick bite, tick removal method, and hospitalization or treatment refer to participants’ lifetime history. A history of tick exposure was reported by 16.0% (47/293) of the participants, while 34.5% (101/293) indicated that a relative or someone in their close environment had experienced a tick bite. Among participants who had been bitten by a tick in lifetime, 46.8% (22/47) reported having the tick removed at a health care facility, and 51.1% (24/47) received outpatient medical care following the tick bite. Participants were asked whether they had ever received any education or information regarding CCHF. This variable included formal or informal education received through health care professionals, media, community health education campaigns, or other sources. With respect to CCHF-related education, 72.7% (213/293) of the participants reported that they had not received any formal training on CCHF. Among those who had received training, health care personnel were identified as the primary source of information (38/80, 47.5%). Additionally, nearly one-quarter of the participants (68/293, 23.2%) were employed in occupations considered to be at risk for CCHF.

**Table 1.** Demographic characteristics of the participants (N=293).

Characteristics				Value	
Sex, n (%)					
Male				120 (41.0)	
Female				173 (59.0)	
Educational level, n (%)					
Illiterate				4 (1.4)	
Literate				2 (0.7)	
Primary school				57 (19.5)	
Secondary school				47 (16.0)	
High school				99 (33.8)	
University				75 (25.6)	
Postgraduate				9 (3.1)	
Marital status, n (%)					
Married				196 (66.8)	
Nonmarried				97 (33.1)	
Place of residence, n (%)					
City center				250 (85.3)	
District				20 (6.8)	
Village or town				23 (7.8)	
Occupation, n (%)					
Housewife				74 (25.3)	
Working for someone else				121 (41.3)	
Self-employed				48 (16.4)	
Retired				8 (2.7)	
Student				30 (10.2)	
Unemployed				12 (4.1)	
Chronic disease, n (%)					
Had				62 (21.2)	
Had not				231 (78.8)	
Tick bite on a relative, n (%)					
Yes				101 (34.5)	
No				192 (65.5)	
Bitten by a tick (lifetime), n (%)					
Yes				47 (16.0)	
No				246 (84.0)	
How a tick was removed (n=47), n (%)					
In the health facility				22 (46.8)	
By relatives and friends				10 (21.3)	
By oneself				15 (31.9)	
Health care use following lifetime tick bite (n=47)^a^, n (%)					
Yes hospitalized				1 (2.1)	
Received outpatient treatment				24 (51.1)	
Did not seek medical care				22 (46.8)	
Receiving CCHF^b^ training, n (%)					
Yes				80 (27.3)	
No				213 (72.7)	
Place of education (n=80), n (%)					
Health professional				38 (47.5)	
Media				27 (33.8)	
Other				15 (18.8)	
Occupation with CCHF risk^c^, n (%)					
Yes				68 (23.2)	
No				225 (76.8)	
Age (years; N=293), mean (SD; range)				38.76 (12.92; 13.00-93.00)	

a Health care usage following lifetime tick bite: This variable was assessed only among participants who reported a lifetime history of tick bites (n=47). It reflects health care usage (outpatient treatment or hospitalization) following the tick bite and does not indicate hospitalization due to confirmed CCHF. The cause of hospitalization beyond the tick bite episode was not assessed.

b CCHF: Crimean-Congo Hemorrhagic Fever.

c Occupation with CCHF risk: Participants were asked whether they considered their own occupation to be associated with an increased risk of CCHF exposure (eg, agricultural workers, livestock breeders and farmers, veterinarians, slaughterhouse workers, forestry workers, and hunters). This variable reflects self-reported occupational risk perception and was not based on a predefined list of occupations.

### Factor Analysis Findings for Construct Validity

Factor analysis was applied to determine the construct validity of the scale. KMO and Bartlett tests were conducted to assess the adequacy of the sample for EFA and the suitability of the data for factor analysis. The KMO value was determined as 0.898, and the Bartlett test result was χ^2^_300_=3659.2 (*P*<.001).

In line with the above findings, the principal components method was applied as an EFA to the 29-item CCHF-DAS. The factor analysis findings of the scale are presented in Table 2. In this analysis, the scale was examined under a single subdimension in accordance with the theoretical structure.

**Table 2.** Factor analysis findings for the Crimean-Congo Hemorrhagic Fever Disease Awareness Scale (CCHF-DAS) (29-Item structure).^a^

Item number	Items	Factor loading
1	CCHF^b^ disease is seen common in the spring and summer months when ticks are active.	0.506
2	CCHF disease is transmitted via bitten infected ticks.	0.459
3	CCHF disease can be transmitted through direct contact with the blood and body fluids of farm animals carrying the CCHF virus.	0.639
4	The disease is caused by an infected tick attaching to an individual.	0.435
5	Shaking hands with someone who has CCHF may cause the disease^c^	-0.008
6	The lethality of the CCHF virus is high.	0.476
7	There is no proven treatment for the virus in CCHF disease.	0.628
8	There is no medical measure such as protective serum or antibiotics to prevent CCHF disease.^c^	0.230
9	CCHF disease is an occupational disease.	0.434
10	CCHF is transmitted from person to person through blood and body fluids.	0.618
11	CCHF virus only causes disease in humans.	0.584
12	CCHF virus can be carried by animals.^c^	0.238
13	Sudden onset of high fever is one of the important symptoms of CCHF disease.	0.715
14	Supportive treatment is given in CCHF disease.	0.577
15	Treatment for CCHF disease is based on symptoms.	0.565
16	The most effective way to prevent CCHF disease is personal protection.	0.678
17	Personal protective equipment should be used during contact with animals, as animals that may carry the CCHF virus do not show any signs of disease.	0.671
18	Light-colored clothing should be worn in risky areas to easily see ticks, which play an important role in CCHF transmission.	0.646
19	People coming from the field should scan their bodies for ticks.	0.763
20	There is no vaccine for CCHF disease.	0.550
21	Ticks should be removed from the place where they are attached to the body with appropriate materials and by experienced people.	0.716
22	Alcohol, oil, and similar products should not be poured on the tick.	0.593
23	The first period of CCHF disease resembles the flu.	0.598
24	In the advanced stages of CCHF disease, bleeding may occur from various parts of the body.	0.501
25	Crushing a tick because of the possibility of causing harm is a wrong practice to protect against CCHF disease.	0.553
26	Symptoms of the disease may appear within 1‐9 days.	0.487
27	The virus is not transmitted by consuming cooked infected animal meat.^c^	0.158
28	People working in agriculture and livestock are at risk of CCHF disease.	0.643
29	Health care workers working in areas where the disease is common are at risk of the disease.	0.705

a Total variance explained (%)=31.153.

b CCHF: Crimean-Congo Hemorrhagic Fever Disease.

c Those excluded (factor loading below 0.30).

As seen in Table 2, when the scale items were examined as a single subdimension, the factor loadings of items 5, 8, 12, and 27 were determined to be below 0.30. Therefore, it was decided to remove these 4 items from the scale and reexamine them. However, they could have higher factor loadings on the second or subsequent components which have not been assessed.

Because the number of items had changed, the KMO and Bartlet tests were readministered. The scale’s KMO value was determined to be 0.912, indicating suitability for principal components analysis and adequacy of the sample size. Similarly, the Bartlett test results (χ^2^_4_=3385.4; *P*<.001) indicate that the data are correlated and suitable for factor analysis. Based on these findings, the principal components method was applied to the 25-item CCHF-DAS as an EFA. The factor analysis findings for the CCHF-DAS are presented in Table 3.

**Table 3.** Factor analysis findings for the Crimean-Congo Hemorrhagic Fever Disease Awareness Scale (CCHF-DAS) (25-item structure).^a^

Item number	Items	Factor loading
1	CCHF^b^ disease is seen common in the spring and summer months when ticks are active.	0.508
2	CCHF disease is transmitted via bitten infected ticks.	0.460
3	CCHF disease can be transmitted through direct contact with the blood and body fluids of farm animals carrying the CCHF virus.	0.640
4	The disease is caused by an infected tick attaching to an individual.	0.437
6	The lethality of the CCHF virus is high.	0.474
7	There is no proven treatment for the virus in CCHF disease.	0.625
9	CCHF disease is an occupational disease.	0.429
10	CCHF is transmitted from person to person through blood and body fluids.	0.616
11	CCHF virus only causes disease in humans.	0.584
13	Sudden onset of high fever is one of the important symptoms of CCHF disease.	0.716
14	Supportive treatment is given in CCHF disease.	0.576
15	Treatment for CCHF disease is based on symptoms.	0.567
16	The most effective way to prevent CCHF disease is personal protection.	0.679
17	Personal protective equipment should be used during contact with animals, as animals that may carry the CCHF virus do not show any signs of disease.	0.671
18	Light-colored clothing should be worn in risky areas to easily see ticks, which play an important role in CCHF transmission.	0.649
19	People coming from the field should scan their bodies for ticks.	0.771
20	There is no vaccine for CCHF disease.	0.547
21	Ticks should be removed from the place where they are attached to the body with appropriate materials and by experienced people.	0.721
22	Alcohol, oil, and similar products should not be poured on the tick.	0.592
23	The first period of CCHF disease resembles the flu.	0.596
24	In the advanced stages of CCHF disease, bleeding may occur from various parts of the body.	0.499
25	Crushing a tick because of the possibility of causing harm is a wrong practice to protect against CCHF disease.	0.552
26	Symptoms of the disease may appear within 1‐9 days.	0.484
28	People working in agriculture and livestock are at risk of CCHF disease.	0.647
29	Health care workers working in areas where the disease is common are at risk of the disease.	0.706

a Total variance explained (%)=35.657.

b CCHF: Crimean-Congo Hemorrhagic Fever Disease.

**Figure 1.**
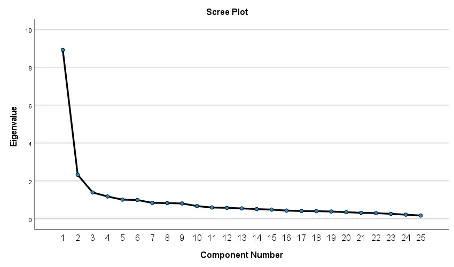
Crimean-Congo Hemorrhagic Fever Disease Awareness Scale (CCHF-DAS) scree plot graph.

As seen in Table 3 and Figure 1, the CCHF-DAS conformed to the theoretical framework with a single-factor structure consisting of 25 items. Factor analysis revealed that all items had factor loadings above 0.30. The total variance explained for the scale was 35.157%. Therefore, no items were removed from the scale at this stage, and the single-dimension, 25-item structure was adopted.

The CCHF-DAS scree plot graph is presented in Figure 1.

The eigenvalues of the CCHF-DAS in the single-factor structure are presented in Figure 1. After the EFA, structural equation modeling was established with CFA to obtain more precise findings.

### CFA Findings

Table 4 presents the fit index values, normal and acceptable values determined for the CCHF-DAS.

**Table 4.** Values of the adaptation index for the Crimean-Congo Hemorrhagic Fever Disease Awareness Scale (CCHF-DAS; normal and acceptable values).

Index	Normal value	Acceptable value	Determined value
χ^2^/SD	<2	<5	4.15
GFI^a^	>0.95	>0.90	0.95
AGFI^b^	>0.95	>0.90	0.94
CFI^c^	>0.95	>0.90	0.97
RMSEA^d^	<0.05	<0.08	0.075
SRMR^e^	<0.05	<0.08	0.072

a GFI: goodness-of-fit index.

b AGFI: adjusted goodness-of-fit index.

c CFI: comparative fit index.

d RMSEA: root mean square error of approximation.

e SRMR: standardized root mean square residual.

As shown in Table 4, several indices were used to examine the fit of the CCHF-DAS model. Of these, the χ^2^/SD value was determined to be 4.15, GFI 0.95, AGFI 0.94, CFI 0.97, RMSEA 0.075, and SRMR 0.072. Some of the relevant fit index values were deemed not to be within the desired range.

**Figure 2.**
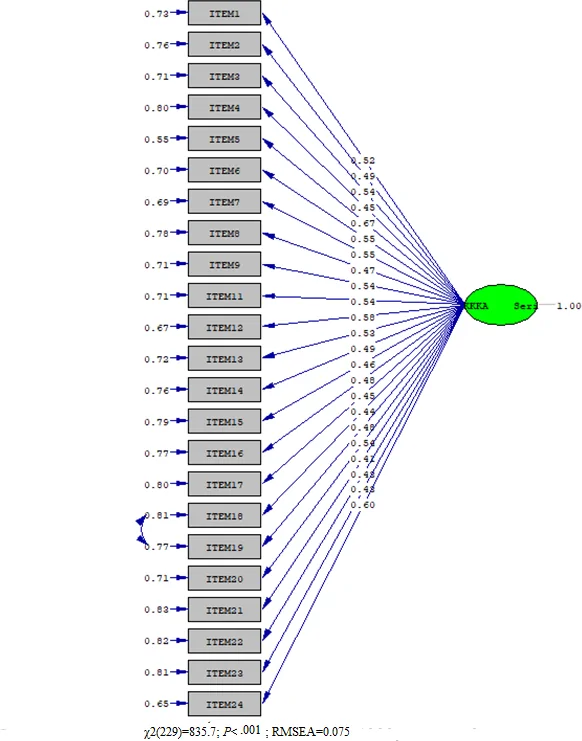
PATH diagram of the Crimean-Congo Hemorrhagic Fever Disease Awareness Scale (CCHF-DAS). RMSEA: root mean square error of approximation.

In Figure 2, factor loadings for the CCHF-DAS are presented as a PATH diagram.

As seen in Figure 2, the factor loadings of the model range from 0.41 to 0.67. The 2-tailed *t*-values of all items in the model are above 1.96 (7.84‐12.74).

### Internal Validity Findings

Table 5 presents the item means, item total correlations, and Cronbach α coefficients if the item is deleted for the CCHF-DAS.

**Table 5.** Item-total correlations and Cronbach α coefficients of the Crimean-Congo Hemorrhagic Fever Disease Awareness Scale (CCHF-DAS).^a^

Item number	Items	Value, n	Mean (SD)	Item-total correlation	If the item is deleted, α
1	CCHF disease is seen common in the spring and summer months when ticks are active.	293	4.29 (0.77)	0.440	0.915
2	CCHF disease is transmitted via bitten infected ticks.	293	4.09 (0.78)	0.397	0.915
3	CCHF disease can be transmitted through direct contact with the blood and body fluids of farm animals carrying the CCHF virus.	293	3.57 (1.28)	0.609	0.912
4	The disease is caused by an infected tick attaching to an individual.	293	4.04 (0.91)	0.381	0.916
6	The lethality of the CCHF virus is high.	293	4.10 (0.90)	0.420	0.915
7	There is no proven treatment for the virus in CCHF disease.	293	3.33 (1.29)	0.606	0.912
9	CCHF disease is an occupational disease.	293	3.39 (1.45)	0.369	0.918
10	CCHF is transmitted from person to person through blood and body fluids.	293	3.54 (1.40)	0.601	0.912
11	CCHF virus only causes disease in humans.	293	3.35 (1.41)	0.567	0.913
13	Sudden onset of high fever is one of the important symptoms of CCHF disease.	293	4.28 (0.70)	0.669	0.912
14	Supportive treatment is given in CCHF disease.	293	3.84 (0.86)	0.523	0.913
15	Treatment for CCHF disease is based on symptoms.	293	3.95 (0.84)	0.506	0.914
16	The most effective way to prevent CCHF disease is personal protection.	293	4.27 (0.78)	0.611	0.912
17	Personal protective equipment should be used during contact with animals, as animals that may carry the CCHF virus do not show any signs of disease.	293	4.20 (0.90)	0.607	0.912
18	Light-colored clothing should be worn in risky areas to easily see ticks, which play an important role in CCHF transmission.	293	4.31 (0.90)	0.579	0.912
19	People coming from the field should scan their bodies for ticks.	293	4.28 (1.11)	0.746	0.909
20	There is no vaccine for CCHF disease.	293	3.30 (1.29)	0.528	0.913
21	Ticks should be removed from the place where they are attached to the body with appropriate materials and by experienced people.	293	4.43 (0.82)	0.660	0.911
22	Alcohol, oil, and similar products should not be poured on the tick.	293	4.03 (1.14)	0.552	0.913
23	The first period of CCHF disease resembles the flu.	293	3.30 (1.08)	0.575	0.912
24	In the advanced stages of CCHF disease, bleeding may occur from various parts of the body.	293	3.59 (0.96)	0.453	0.914
25	Crushing a tick because of the possibility of causing harm is a wrong practice to protect against CCHF disease.	293	4.04 (0.97)	0.494	0.914
26	Symptoms of the disease may appear within 1‐9 days.	293	3.73 (0.90)	0.429	0.915
28	People working in agriculture and livestock are at risk of CCHF disease.	293	4.27 (0.84)	0.571	0.913
29	Health care workers working in areas where the disease is common are at risk of the disease.	293	4.21 (0.84)	0.642	0.912

a Total Cronbach α=0.916.

As shown in Table 5, the total Cronbach α coefficient for the CCHF-DAS is 0.916. Total item correlation values for all items on the scale are above 0.30, and deleting any item does not cause a significant increase in the Cronbach α coefficient. Therefore, no items were removed from the scale at this stage.

For the internal consistency reliability coefficient of the CCHF-DAS, the scale was divided into 2 halves, and the consistency values for the 2 halves are given in Table 6.

**Table 6.** Split-half reliability values of the Crimean-Congo Hemorrhagic Fever Disease Awareness Scale (CCHF-DAS).

Split-half reliability			Values
Cronbach α			
First half			
Value			0.833
Number of items^a^			13
Second half			
Value			0.868
Number of items^b^			12
Total number of items			25
Correlation between the 2 halves			0.816
Spearman-Brown coefficient			
Equal length			0.899
Unequal length			0.899
Guttman Split-Half coefficient			0.898

a The items are Item1, Item2, Item3, Item4, Item5, Item6, Item7, Item8, Item9, Item10, Item11, Item12, Item13.

b The items are: Item14, Item15, Item16, Item17, Item18, Item19, Item20, Item21, Item22, Item23, Item24, Item25.

An examination of Table 6 revealed that the split-half reliability values for the internal consistency of the CCHF-DAS were found to be at acceptable levels. This value was 0.833 for the first half of the 13-item scale and 0.868 for the second half, consisting of 12 items. The results indicated that the correlation between the 2 halves was 0.816, the Spearman-Brown coefficient was 0.899, and the Guttman Split-Half coefficient was 0.898, indicating that the scale had good internal consistency reliability. These findings indicate that the CCHF-DAS has high internal consistency reliability.

Convergent and divergent validity analysis results are presented in Table 7.

**Table 7.** Convergent and divergent validity analysis results.

Subdimensions	CR^a^	AVE^b^	Omega
Crimean-Congo Hemorrhagic Fever Disease Awareness Scale	0.931	0.356	0.931
Expected values	≥0.70	≥0.50	≥0.70

a CR: composite reliability.

b AVE: average variance extracted.

In the convergent-divergent validity analysis, CR and AVE values were examined. CR values were 0.931 and ranged from acceptable to excellent reliability. AVE was 0.356 and below 0.50. Additionally, McDonald ω values were 0.931 and ranged from acceptable to excellent.

The distribution of minimum, maximum, and mean scores obtained from the CCHF-DAS are 37.00, 125.00, and 125, respectively.

Participants received a total score of 97.75 (SD 14.82) on the CCHF Awareness Scale.

## Discussion

### Interpretation of the Findings

The primary objective of this study was to develop and psychometrically evaluate a valid and reliable instrument for measuring adults’ awareness of CCHF. This objective was achieved through a systematic scale development process involving content validation, pilot testing, EFA and CFA, and multiple reliability assessments. The final 25-item, unidimensional CCHF-DAS demonstrated satisfactory construct validity, excellent internal consistency, and acceptable model fit indices, supporting its use as a standardized instrument for assessing CCHF awareness in adult populations.

### Principal Findings

Studies on CCHF in the literature generally consist of cross-sectional investigations evaluating knowledge, attitudes, and practices levels among various risk groups, such as the general public, livestock value chain actors, medical and pharmacy students, health care personnel, or slaughterhouse workers [18-24]. Accurately measuring societal awareness and risk perception in combating CCHF, which possesses an endemic character in Turkey and worldwide, is of vital importance for the development of preventive health policies. However, a review of the literature reveals a notable lack of a psychometrically robust instrument specifically measuring CCHF awareness in the adult population. The CCHF-DAS developed in this study makes an important contribution to the literature by filling this methodological gap. The stages followed in the scale development process [25], data analysis techniques [26,27], and validation processes [12-15,28-31] were conducted in accordance with international scale development standards.

As a result of the EFA conducted to examine the construct validity of the scale, it was determined that the items had factor loadings above 0.30, and the scale exhibited a single-factor structure consisting of 25 items. The total variance explained by the single-factor structure was found to be 35.157%. Following the EFA, the fit indices (χ^2^/SD=4.15; GFI=0.95; AGFI=0.94; CFI=0.97, RMSEA=0.075; and SRMR=0.072) obtained from the CFA established to validate the model were examined. Although some fit indices were at borderline values, they were generally observed to be within acceptable limits. It was determined that the factor loadings of the items in the model ranged from 0.41 to 0.67, and the *t*-values of all items were above 1.96 (ranging between 7.84 and 12.74).

The KMO value of 0.912 and a statistically significant Bartlett test of sphericity (*P*<.001) indicated that the sample size and data structure were highly suitable for factor analysis [11,25]. These results reflect strong inter-item correlations and support the adequacy of the dataset for psychometric evaluation.

Although items 5, 8, 12, and 27 were removed from the final scale because of low factor loadings, their weak association with the overall awareness construct may reflect inconsistent or insufficient knowledge regarding specific aspects of CCHF transmission, animal reservoirs, and prevention. Therefore, these items may still provide useful insight into community-level misconceptions and should be considered when planning targeted health education strategies.

Although the Kaiser criterion initially suggested more than one factor with eigenvalues greater than 1.0, factor retention was not determined solely on the basis of the eigenvalue criterion. The scree plot indicated a clear inflection after the first factor, suggesting the presence of 1 dominant underlying construct. Moreover, the CCHF-DAS was theoretically developed to assess overall disease awareness as a single latent construct, with items covering transmission, symptoms, prevention, and treatment as complementary components of the same construct rather than independent dimensions. Therefore, considering the theoretical framework, the scree plot, and the principle of parsimony, a unidimensional model was retained. This decision was further supported by the CFA, which demonstrated acceptable model fit indices and excellent internal consistency for the final 25-item scale [26,27].

In our study, the total Cronbach α coefficient calculated to evaluate the internal consistency reliability of the scale was found to be 0.916. The item-total correlations being above 0.30 and the removal of any item not causing a significant increase in the Cronbach α coefficient indicate that all items in the scale contribute homogeneously to the structure. Furthermore, within the scope of split-half reliability, the values were determined as 0.833 for the first half, 0.868 for the second half, with a correlation of 0.816 between the 2 halves, a Spearman-Brown coefficient of 0.899, and a Guttman Split-Half coefficient of 0.898, proving that the CCHF-DAS possesses a high level of internal consistency reliability. In the convergent and discriminant validity analysis, the CR value was found to be 0.931 (at an acceptable and excellent level), while the AVE value remained below 0.50 at 0.356. Similarly, McDonald Ω value was calculated as 0.931. Reliability analyses demonstrated excellent internal consistency of the CCHF-DAS. The Cronbach α coefficient for the overall scale was 0.916, exceeding the recommended threshold for high reliability [25]. In addition, split-half reliability coefficients and other internal consistency indices confirmed the stability and consistency of the scale items [14,28-31]. These findings suggest that the CCHF-DAS provides reliable measurements of disease awareness across items and respondents.

Although the AVE value (0.356) was below the recommended threshold of 0.50, the CR value of 0.931 indicated excellent construct reliability. As suggested by Fornell and Larcker [14], convergent validity may still be acceptable when CR exceeds 0.60. Moreover, Hair et al [26] recommend evaluating convergent validity using multiple complementary indicators, including factor loadings, CR, AVE, and overall model fit, rather than relying solely on AVE [26]. Nevertheless, the relatively low AVE should be considered a limitation, and future studies should further evaluate and refine the scale using independent samples.

Because the data were collected using self-report questionnaires administered at a single point in time, the potential influence of CMV was considered. The Harman single-factor test indicated that the first unrotated factor accounted for 35.66% of the total variance, which is below the commonly accepted threshold of 50%. This finding suggests that common method bias is unlikely to have substantially affected the observed relationships. Nevertheless, as with all self-report measures, the possibility of residual method bias cannot be completely excluded. Future studies should further minimize this potential limitation by incorporating multiple data sources, longitudinal designs, or procedural and statistical remedies for CMV.

Participants obtained a mean total score of 97.75 (SD 14.82) on the CCHF-DAS. This score indicates that while the target population possesses a certain level of awareness regarding CCHF, there remains an ongoing need for sustainable educational and intervention programs across the general public. Previous studies in the literature have examined individuals’ knowledge, attitudes, and practices related to CCHF; however, these studies predominantly relied on author-generated questionnaires rather than standardized measurement instruments [18-24]. As a result, comparisons across studies and populations have been limited. To the best of our knowledge, the CCHF-DAS is the first validated and reliable scale that systematically assesses awareness of CCHF by addressing the causative agent, transmission routes, clinical manifestations, treatment, and preventive measures within a single measurement framework.

National and international studies have consistently reported insufficient levels of knowledge and preventive practices related to CCHF and have emphasized the need for comprehensive strategies to control the disease [18-24]. The findings of the present study indicate that although participants demonstrated an overall acceptable level of awareness, there remains a substantial need to further enhance awareness, particularly given the ongoing disease burden, high mortality rates, and absence of a specific antiviral treatment or licensed vaccine. Assessing disease awareness using a validated instrument such as the CCHF-DAS may facilitate the identification of knowledge gaps and inform the development of targeted public health interventions aimed at promoting preventive behaviors and early health care seeking.

### Limitations

While this study provides a robust and psychometrically sound instrument for assessing CCHF awareness, several limitations must be acknowledged to contextualize the findings appropriately:

- Geographic and sampling constraints: The study was conducted within a single geographic region, with participants recruited exclusively through family health centers. Consequently, the generalizability of the findings to broader populations, diverse demographic groups, or individuals residing in nonendemic or distinct socioeconomic settings remains limited. Future research should validate the CCHF-DAS across multicenter, nationwide, and culturally diverse cohorts.
- Cross-sectional design: The cross-sectional nature of the study precludes any causal inferences regarding the directionality of the relationships between influencing factors and CCHF awareness. Longitudinal designs are warranted to track how awareness evolves over time and to evaluate the predictive validity of the scale regarding actual protective behaviors.
- Absence of temporal reliability metrics: Because the scale was administered for the first time in this study, test-retest reliability and sensitivity to change over time could not be evaluated. Subsequent longitudinal investigations should assess the stability and responsiveness of the CCHF-DAS following targeted educational interventions.
- Methodological approach to factor analysis: Both EFA and CFA were performed using the same sample dataset. Although this practice appears in scale development literature, relying on a single sample increases the risk of overfitting. Future studies should test and confirm the factor structure using independent, newly drawn samples to establish stronger evidence of structural stability and external validity.

### Conclusions

The CCHF-DAS, developed in this study, is a novel and psychometrically robust instrument capable of reliably and validly measuring CCHF awareness among adult populations, thereby filling a significant methodological gap in the literature. The findings demonstrate that the structural and internal consistency properties of the scale largely meet internationally accepted standards, confirming its utility for public health research. Beyond providing a psychometric measurement tool, the broader implications of this study offer critical insights for comprehensive public health policies and preventive medicine strategies:

- Epidemiological surveillance and risk stratification: The ability to monitor the awareness levels of adult populations residing in endemic regions using a standardized scale enables public health authorities to identify at-risk groups with greater precision. This facilitates the rational allocation of limited health resources, directing them toward the most vulnerable segments of the population.
- Evidence-based health education and intervention programs: The total mean scores obtained in this study and the detailed feedback provided by the scale items offer a concrete baseline to enhance the efficacy of community-based educational campaigns. Rather than relying on uniform and traditional informational strategies, the scale guides the design of dynamic intervention programs that directly target individuals’ knowledge gaps and misconceptions.
- Interdisciplinary and sustainable control: Combating vector-borne zoonotic diseases such as CCHF requires coordinated efforts across medicine, veterinary science, environmental health, and the social sciences. The developed scale can serve as a common metric for evaluating the effectiveness of field studies conducted by various stakeholders, while also providing empirical data support for future epidemiological modeling.

In conclusion, the data generated through the CCHF-DAS should serve as a core component of strategic planning aimed at mitigating societal vulnerabilities, extending far beyond the mere measurement of individual awareness. Future longitudinal and independent sample validation studies across diverse geographical and cultural contexts will reinforce the universal validity of this scale and add significant value to global zoonotic disease management.

### Public Health Implications

CCHF remains a significant public health challenge due to its high mortality rate, expanding geographic distribution, and the absence of a specific antiviral treatment or licensed vaccine. In this context, prevention and early detection are critically dependent on individual and community-level awareness. The CCHF-DAS provides a practical and evidence-based tool for assessing awareness levels and identifying knowledge gaps related to CCHF among adults.

The use of the CCHF-DAS in public health practice may support the planning, implementation, and evaluation of targeted educational and preventive interventions, particularly for high-risk groups such as agricultural workers, livestock handlers, health care workers, and individuals living in endemic regions. By enabling the systematic measurement of awareness, the scale can help guide resource allocation, tailor risk communication strategies, and monitor the effectiveness of awareness-raising programs over time. Ultimately, improving individual awareness of CCHF through informed public health interventions may contribute to reducing disease transmission, morbidity, and mortality at the population level.

## Supplementary material

10.2196/93724 Multimedia Appendix 1 Turkish version of the scale.

## Abbreviations

AGFI: adjusted goodness-of-fit index
AVE: average variance extracted
CCHF: Crimean-Congo Hemorrhagic Fever
CCHF-DAS: Crimean-Congo Hemorrhagic Fever Disease Awareness Scale
CFA: confirmatory factor analysis
CFI: comparative fit index
CMV: common method variance
CR: composite reliability
EFA: exploratory factor analysis
GFI: goodness-of-fit index
I-CVI: Item Content Validity Index
KMO: Kaiser-Meyer-Olkin
RMSEA: root mean square error of approximation
SRMR: standardized root mean square residual
STROBE: Strengthening the Reporting of Observational Studies in Epidemiology
