# Supplementary material for: Crimean-Congo Hemorrhagic Fever Disease Awareness Scale: Scale Development, Validity, and Reliability Study
Source: JMIR Public Health Surveill. 2026 Sep 14;12:e93724. doi: 10.2196/93724 (PMC13574330; doi:10.2196/93724)
Supplement: Supplementary file 1 [file publichealth-v12-e93724-s001.docx]

**Multimedia Appendix 1**

**TURKISH VERSION OF THE SCALE**

**KIRIM KONGO KANAMALI ATEŞİ HASTALIĞI FARKINDALIĞI ÖLÇEĞİ (KKKA-HFÖ)**

| **No** | **KKKA-HFÖ** | **Hiç katılmıyorum** | **Katılmıyorum** | **Kararsızım** | **Katılıyorum** | **Kesinlikle katılıyorum** |
| --- | --- | --- | --- | --- | --- | --- |
| **1** | KKKA hastalığı, kenelerin aktif olduğu ilkbahar ve yaz aylarında sık görülür |  |  |  |  |  |
| **2** | KKKA hastalığı enfekte kenelerin tutması sonucu bulaşır |  |  |  |  |  |
| **3** | KKKA hastalığı KKKA virüsünü taşıyan çiftlik hayvanının kan ve vücut sıvıları ile doğrudan temas sonucu bulaşabilir |  |  |  |  |  |
| **4** | Enfekte kenenin bireyi tutması hastalık sebebidir |  |  |  |  |  |
| **5** | KKKA virüsünün öldürücülük gücü yüksektir |  |  |  |  |  |
| **6** | KKKA hastalığında virüse yönelik etkisi kanıtlanmış bir tedavisi yoktur |  |  |  |  |  |
| **7** | KKKA hastalığı bir meslek hastalığıdır |  |  |  |  |  |
| **8** | KKKA hastalığı kan ve vücut sıvılarıyla insandan insana bulaşır |  |  |  |  |  |
| **9** | KKKA virüsü sadece insanlarda hastalık yapar |  |  |  |  |  |
| **10** | Ani başlayan yüksek ateş KKKA hastalığının önemli belirtilerinden biridir |  |  |  |  |  |
| **11** | KKKA hastalığında destekleyici tedavi yapılır |  |  |  |  |  |
| **12** | KKKA hastalığında belirtilere yönelik tedavi yapılır |  |  |  |  |  |
| **13** | KKKA hastalığını önlemede en etkili yol kişisel korunmadır |  |  |  |  |  |
| **14** | KKKA virüsünü taşıma ihtimali olan hayvanların hastalık belirtisi vermemesi nedeniyle hayvanlarla temas sırasında kişisel koruyucu ekipman kullanılmalıdır |  |  |  |  |  |
| **15** | KKKA bulaşında önemli rol oynayan kenelerin rahat görülmesi için riskli alanlarda açık renkli kıyafetler giyilmelidir |  |  |  |  |  |
| **16** | Araziden gelen kişiler vücutlarında kene taraması yapmalıdır |  |  |  |  |  |
| **17** | KKKA hastalığının aşısı yoktur |  |  |  |  |  |
| **18** | Keneler vücuda tutunduğu yerden uygun malzemeyle ve deneyimli kişiler tarafından çıkarılmalıdır |  |  |  |  |  |
| **19** | Kenenin üzerine alkol, sıvı yağ ve benzeri ürünler dökülmemelidir |  |  |  |  |  |
| **20** | KKKA hastalığının ilk dönemi gribe benzemektedir |  |  |  |  |  |
| **21** | KKKA hastalığının ileri döneminde vücudun çeşitli bölgelerinden kanamalar oluşabilir |  |  |  |  |  |
| **22** | Bir kenenin zarar verme ihtimali düşünülerek ezilmesi KKKA hastalığından korunmaya yönelik yanlış bir uygulamadır |  |  |  |  |  |
| **23** | Hastalık belirtileri 1-9 gün arasında ortaya çıkabilir |  |  |  |  |  |
| **24** | Tarım ve hayvancılıkla ilgili işlerde çalışanlar KKKA hastalığı açısından risk altındadır |  |  |  |  |  |
| **25** | Hastalığın sık görüldüğü bölgede çalışan sağlık personeli hastalık açısından risk altındadır |  |  |  |  |  |
